# Dysregulation of Intestinal Epithelial Cell RIPK Pathways Promotes Chronic Inflammation in the IBD Gut

**DOI:** 10.3389/fimmu.2019.01094

**Published:** 2019-05-20

**Authors:** Ricard Garcia-Carbonell, Shih-Jing Yao, Soumita Das, Monica Guma

**Affiliations:** ^1^Department of Pathology, University of California, San Diego, San Diego, CA, United States; ^2^Medicine, School of Medicine, University of California, San Diego, San Diego, CA, United States

**Keywords:** IBD, apoptosis, RIPK, A20, RIPK1, NOD2, autophagy

## Abstract

Crohn's disease (CD) and ulcerative colitis (UC) are common intestinal bowel diseases (IBD) characterized by intestinal epithelial injury including extensive epithelial cell death, mucosal erosion, ulceration, and crypt abscess formation. Several factors including activated signaling pathways, microbial dysbiosis, and immune deregulation contribute to disease progression. Although most research efforts to date have focused on immune cells, it is becoming increasingly clear that intestinal epithelial cells (IEC) are important players in IBD pathogenesis. Aberrant or exacerbated responses to how IEC sense IBD-associated microbes, respond to TNF stimulation, and regenerate and heal the injured mucosa are critical to the integrity of the intestinal barrier. The role of several genes and pathways in which single nucleotide polymorphisms (SNP) showed strong association with IBD has recently been studied in the context of IEC. In patients with IBD, it has been shown that the expression of specific dysregulated genes in IECs plays an important role in TNF-induced cell death and microbial sensing. Among them, the NF-κB pathway and its target gene TNFAIP3 promote TNF-induced and receptor interacting protein kinase (RIPK1)-dependent intestinal epithelial cell death. On the other hand, RIPK2 functions as a key signaling protein in host defense responses induced by activation of the cytosolic microbial sensors nucleotide-binding oligomerization domain-containing proteins 1 and 2 (NOD1 and NOD2). The RIPK2-mediated signaling pathway leads to the activation of NF-κB and MAP kinases that induce autophagy following infection. This article will review these dysregulated RIPK pathways in IEC and their role in promoting chronic inflammation. It will also highlight future research directions and therapeutic approaches involving RIPKs in IBD.

Inflammatory bowel disease (IBD) is an inflammatory process with a chronic relapsing course that is characterized pathologically by intestinal inflammation and epithelial injury that affects the different gastrointestinal (GI) linings (1). IBD includes different inflammatory pathologies of the gastrointestinal track. The more prevalent IBD pathologies are Crohn's Disease (CD) and Ulcerative Colitis (UC) (1). Pathogenesis of IBD is multifactorial, involving genetic predisposition, disturbance of the commensal microbiota, epithelial barrier defects, dysregulated immune responses, and environmental factors (2). The gastrointestinal tract (in particular, the terminal ileum and colon) also contains a massive bacterial load that has the potential to initiate an acute inflammatory intestinal response if the mucosal barrier is breached and bacteria gain access to the lamina propria, as occurs in IBD (2).

The receptor interacting protein kinase (RIPK) proteins are key molecules for the maintainance of a healthy intestinal barrier (3). The RIPK family contains seven members that share a homologous serine-threonine kinase domain but has different functional domains (4). RIPK1 contains a death-domain in the C-terminal portion that allows its recruitment to different signaling complexes. RIPK2 is characterized by its caspase activation and recruitment domain (CARD). RIPK3, like RIPK1, has a RIP homotypic interaction motif (RHIM), which is necessary for RIPK1 and RIPK3 dimerization. RIPK4 (or DIK or PKK) and RIPK5 (or SgK288) contain ankyrin repeats in the C-terminal tail. Finally, RIPK6 (or LRRK1) and RIPK7 (LRRK2) have leucine-rich repeats (LRR) that could play a role in the recognition of inflammatory-associated molecular patterns. In this review, we will focus on the epithelial barrier and how an aberrant response to TNF stimulation, exarcebated, IBD-associated microbial sensing, and abnormal regeneration and healing of the injured mucosa by dysregulated RIPK pathways in IEC can critically affect the health of the intestinal barrier.

## Intestinal Epithelial Barrier

**Figure 1:**
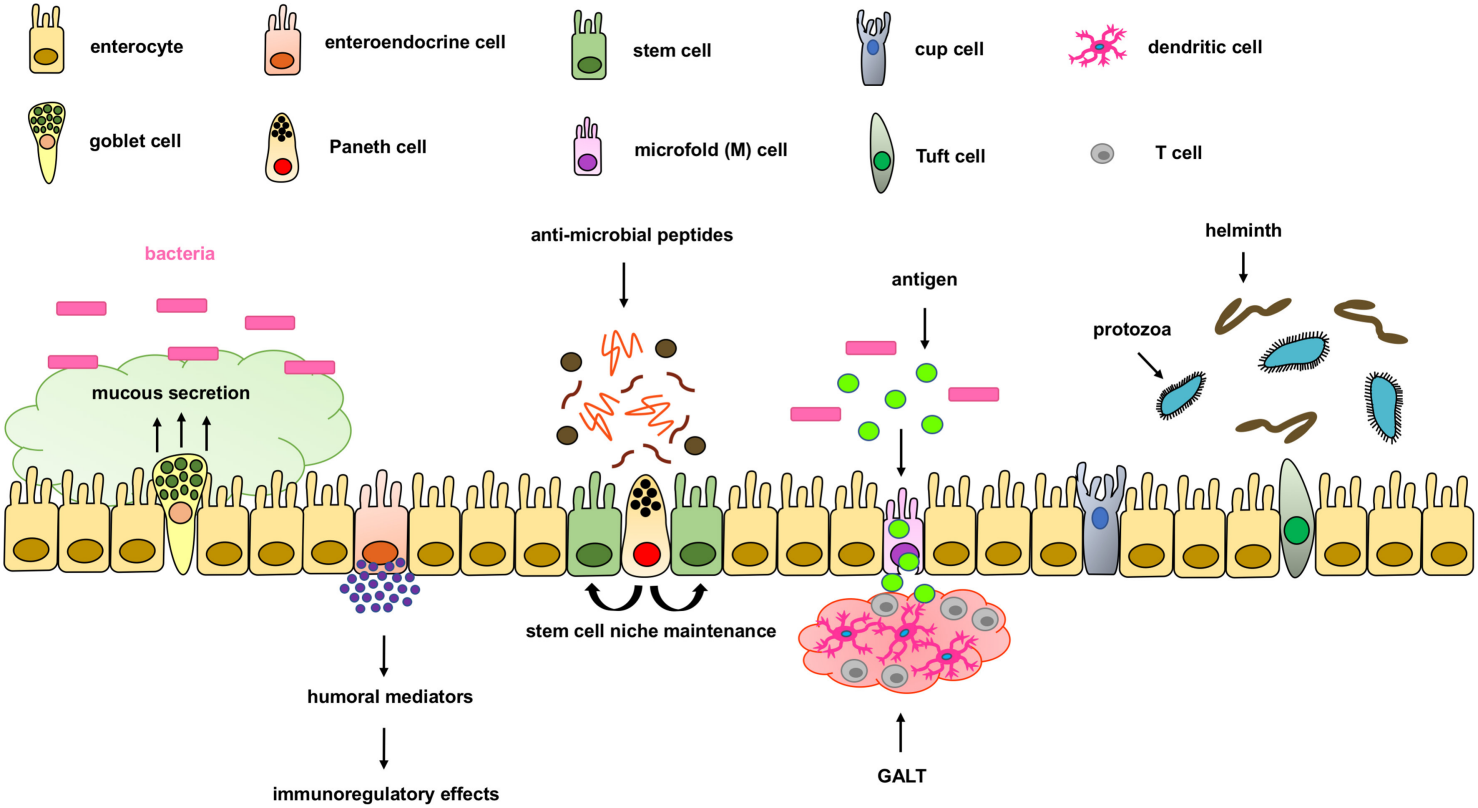
Components of the intestinal epithelial cell barrier. In the intestinal epithelial cell barrier we can find different specialized cell types including; enterocytes, goblet cells, enteroendocrine cells, paneth cells, stem cells, microfold (M) cells, and cup and tuft cells. Briefly, enterocytes are the most common cells with nutrient absorption functions; goblet cells secrete mucus to form an extra intestinal protective layer; enteroendocrine cells secrete humoral mediators with immunoregulatory effects; paneth cells secrete antimicrobial peptides and maintain the stem cell niche; microfold cells play an important role in transporting lumen antigens into the associated intestinal lymphoid structures; and Tuft cells play an important innate role against helminth and protozoan infections.

The intestinal epithelium forms the physical, protective, and host defense barrier against the harmful luminal microenvironment, while providing selective permeability for absorption of nutrients (5). The epithelium is covered by a single-cell layer composed of different subtypes of specialized intestinal epithelial cells (IECs) including enterocytes, goblet cells, enteroendocrine cells, Paneth cells, M cells, cup cells, and Tuft cells, all of which differentiate from common epithelial stem cells (5). These IECs types are functionally different and essential for maintaining intestinal homeostasis by separating the intestinal lumen from the underlying lamina propria and by controlling the crosstalk between luminal microbiota and subjacent immune cells (Figure 1). IECs not only function as a physical barrier through *enteroctyes* (the largest cell population in IECs), but also through other specific functions. *Paneth cells*, for instance, are specialized secretory epithelial cells located in the crypt of the small intestine and contribute to the host defense secreting anti-microbial peptides that are diluted in the mucus enhancing the antimicrobial barrier and shape the commensal bacterial population (6–9). Paneth cells are characterized by an extensive endoplasmatic reticulum and Golgi apparatus with big secretory granules containing a wide variety of peptides, especially those with antimicrobial activity including defensins.

*Goblet cells* are the second most abundant cells in IECs and are specialized in mucus secretion (10). Mucins are highly O-glycosylated molecules that have gel-like properties and cover the inner walls of the gut lumen. Mucins form a bistratified mucus barrier, which becomes denser as it nears IECs, thus preventing bacteria from penetrating the barrier (11). At the same time, the mucus provides digestible glycans as a stable source of energy for the commensal microbiome (12–14). Intestinal goblet cells also sense luminal material that can be taken up delivered to lamina propria CD103+CD11c+ dendritic cells (DC) (15, 16) through goblet cell-associated antigen passages (GAPs). The DCs that interact with regulatory T cells have been suggested to induce tolerance to food antigens. Other cells, such as *enteroendocrine cells*, release a variety of humoral and paracrine mediators that can induce different immunoregulatory effects including cellular recruitment, activation, phagocytosis, antigen presentation and cytokine secretion (17, 18). Additionally, *tuft cells*, critical in the initiation of type-2 immune responses, are typically activated during intestinal protozoa or helminth parasite infections. *Microfold (M) cells* are epithelial cells specialized in phagocytosis and transcytosis of gut lumen antigens and pathogenic or commensal microorganisms across the intestinal epithelium toward the underlying gut-associated lymphoid tissues (GALT). M cells are also critical in maintaining a healthy intestinal barrier and control the crosstalk between luminal microbiota and subjacent immune cells.

IECs ability to act as a protective physical barrier is mediated by the formation of protein complex connections between adjacent cells, including tight junctions (TJ) and adherent junctions (AJ), which form the apical junction complex (AJC), as well as desmosomes, which are located in the basolateral membrane (19). These dynamic complexes are susceptible to endogenous and exogenous factors, such as cytokines, nutrients, and bacteria (19). TJs are the apical complexes of the AJC, connecting and sealing adjacent cells. TJ complexes are composed of junctional adhesion molecules (JAM), claudins, occludins, and zonula occludens (ZO), which seal neighboring cells together (20). AJs, composed of cadherins, form the second AJC loop, maintaining cell-to-cell connections; however, AJ are not critical for creating paracellular tightness (20). Finally, desmosomes connect intermediate filaments of neighboring cells, conferring mechanical strength to cell-to-cell junctions. They are formed by desmoplakin, plakoglobin, plakophilin, desmocollin, and desmoglein (21, 22). Tight junctions are critical for maintaining barrier function during IEC shedding, which occurs continuously from villus tips or colonic surfaces as a result of migration of the epithelial cell up the crypt–villus axis from stem cells at the base of the crypt (23). Normal cell shedding never causes a breach in the epithelial barrier because of the redistribution of tight junction proteins that facilitates the closure of the gap (24). However, in pathological conditions, when multiple neighboring cells are shed at the same time or cell death is activated, or turnover is increased a proper rearrangement of cell-to-cell contact cannot take place. Consequently, breaches appear in the intestinal epithelial barrier, which causes intestinal inflammation (23).

## RIPK Proteins are Critical to Maintainance of Barrier Function

### The Role of Autophagy Mediated by Nod2/RIPK2 in Maintaining Intestinal Homeostasis

Autophagy is a cell stress response that causes the encapsulation of cellular contents for subsequent degradation and recycling (25). Although the first barrier against bacterial and parasitic invasion of the intestine is the mucus layer, some pathogens can penetrate this layer to reach the IECs. In this situation, autophagy plays an important role by recognizing and degrading intracellular pathogens, thus functioning as an innate barrier to infection. It has already been shown that knockdown of autophagy genes in *Caenorhabditis elegans* and *Dictyostelium discoideum* increases *Salmonella typhimurium* intracellular replication, decreases animal lifespan, and results in apoptotic-independent death (26).

NOD2 (nucleotide-binding oligomerization domain-containing protein 2) is a critical element in regulating autophagy in IECs (27). NOD2, a cytosolic pattern recognition receptor, is activated by the peptidoglycan fragment muramyl dipeptide (MDP) to generate a proinflammatory immune response (28, 29). Over 30 cellular proteins interact with NOD2 directly and influence or regulate its functional activity (30). Among them, NOD2 recruits ATG16L1 (autophagy-related protein 16 like 1) to the plasma membrane at the bacterial entry site to induce phagophore formation. ATG16L1 then forms a complex with ATG5 and ATG12 to induce the lipidation of LC3 (microtubule-associated protein 1A/1B-light chain 3), forming an autophagosome and inducing autophagy (27). Additionally, upon activation, NOD1 and NOD2 recruit RIPK2 through CARD domains (31, 32), inducing RIPK2 k63-polyubiquitination in lysine 209 by cIAPs and the LUBAC complex (33–35). This leads to RIPK2 activation, which depends on autophosphorylation in residues Ser176 and Tyr474, an essential and enhancing site respectively (36, 37), and downstream activation of transforming growth factor beta-activated kinase 1 (TAK1) (38–40). TAK1 consecutively phosphorylates the IKK complex triggering NF-κB and MAPK pathway activation.

Travassos et al. showed that NOD1 and NOD2 can recruit ATG16L1 to the plasma membrane at the bacterial entry site in different cell types including the mouse intestinal epithelial cell line Mode-K through a RIPK2-independent mechanism (41). The role of RIPK2 as a kinase in autophagy induction downstream of NOD2 has also been investigated. In different cells, including the cell-like HCT116, RIPK2 kinase function is required for the phosphorylation of the protein kinase ULK1 at Ser555, and for the deactivation of the protein phosphatase 2A (PP2A) complex that negatively regulates autophagy induction downstream of p38 activation (42, 43). In dendritic cells, NOD2 is also able to trigger autophagy through RIPK2-mediated recruitment of ATG5, ATG7, and ATG16L1 (44). Anand et al. showed how activated RIPK2 promotes increased autophagosome formation by activating MAPK/ERK kinase 4 (MEKK4)–p38 signaling and/or extracellular signal-regulated kinase 1 (ERK1) and ERK2 signaling, which upregulates basal levels of autophagy (43).

Autophagy also plays an important role in protecting IECs from cell death. NOD2 is highly expressed in intestinal stem cells, and its activation by MDP triggers stem cell survival and strong cytoprotection against oxidative stress-mediated cell death (45). This could be due to NOD2s ability to activate the NF-κB pathway, which has protective effects in the intestinal epithelium (46). Animals lacking ATG16L1 in the epithelium were more susceptible to DSS-induced colitis, and the pathology was exacerbated when these animals were infected with murine norovirus (MNV). Further histological analysis and organoid experiments show that ATG16L1 protects cells from necroptosis by removing aberrant mitochondria and impairing downstream reactive oxygen species (ROS) accumulation (47). In another model of colitis induced by *Helicobacter hepaticus*, mice with a deletion of ATG16L1 in IECs had worse histopathology than their littermates. IECs in affected mice were more susceptible to TNF-induced apoptotsis, increasing inflammation and pathology of the models (48). Overall, it has been shown that autophagy adds another layer of protection from foreign organisms by preventing pathogen proliferation and dissemination to extraintestinal sites (49). It also has a protective role in IECs, and defects in the autophagy pathway increase the susceptibility of the intestine to inflammation, inducing cell death and intestinal epithelial barrier breakdown.

## RIPK1 and RIPK3 are Critical in Maintaining an Equilibrium Between Cell Survival and Cell Death Downstream of TNF

### RIPK1 and RIPK3

**Figure 2:**
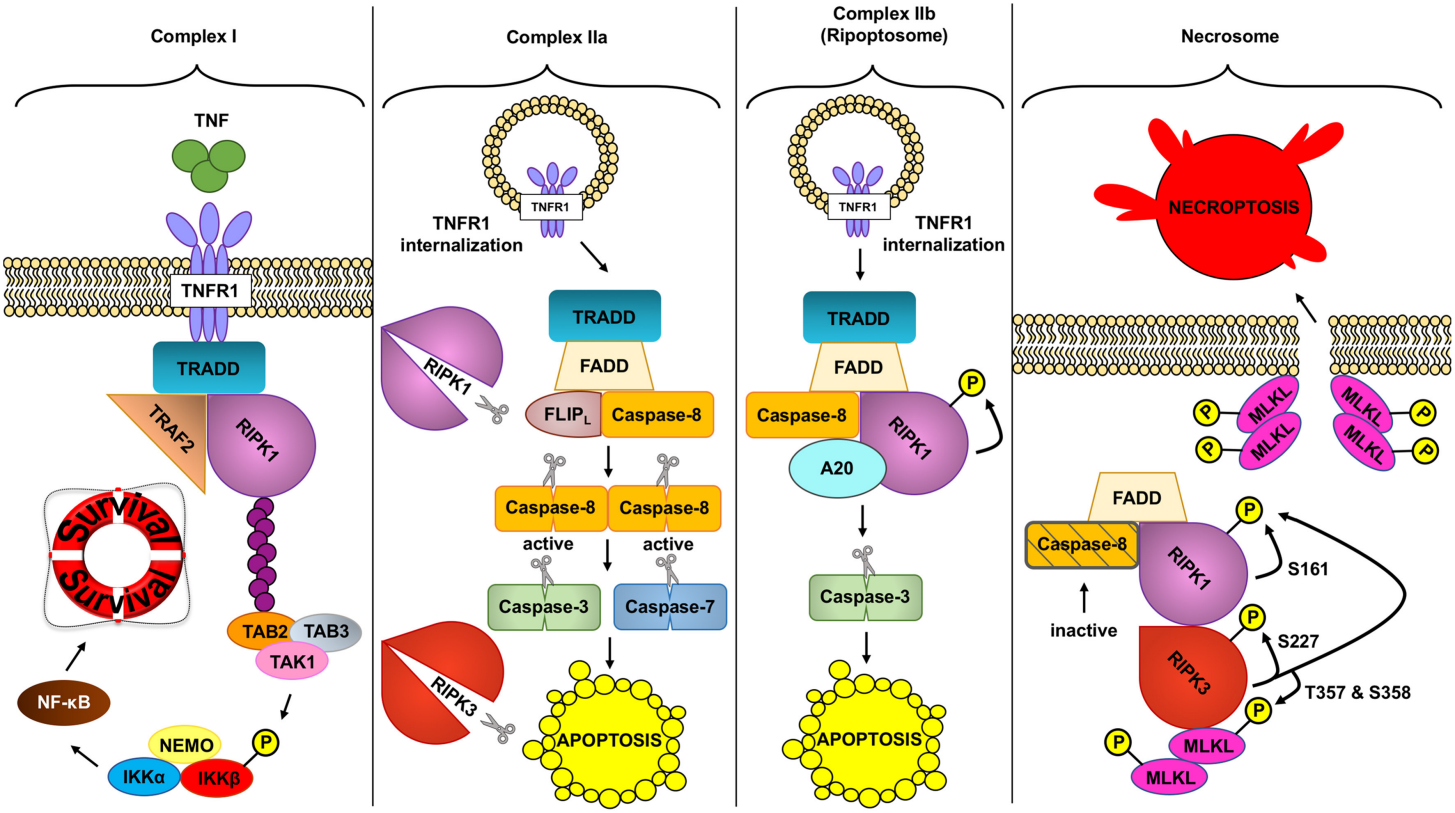
Different TNFR complex formed downstream of TNF. TNF (tumor necrosis factor) can induce the formation of different complexes with diverse outcomes depending on the conditions. In a homeostatic situation, TNF triggers the formation of complex I, where RIPK1 (receptor interacting protein kinase 1) acts as a scaffold protein allowing the activation of the pro-inflammatory and protective pathway NF- κB (nuclear factor kappa-light-chain-enhancer of activated B cells). In pathologic conditions, complex IIa can be formed leading to the activation of caspase-8. Active caspase-8 cleaves RIPK1 and RIPK3 (receptor interacting protein kinase 3) and downstream caspases to induce apoptosis. Unlike complex IIa, complex IIb depends on the kinase role of RIPK1 to activate caspase-8 and execute apoptosis. Inhibition of RIPK1 kinase and ROS (reactive oxygen species) prevents this type of cell death. Finally, if caspases are inhibited and the NF- κB pathway is not activated, TNF can trigger the formation of the necrosome. This complex depends on the kinase activity of RIPK1 and RIPK3 to activate MLKL (mixed lineage kinase domain like). MLKL in turn will create the necroptotic pore in the plasma membrane inducing necroptosis, a regulated type of necrosis.

Two RIPK proteins have key kinase-dependent functions in deciding beneficial or deleterious effects downstream of TNF: RIPK1 and RIPK3 are two key molecules in the assembly of TNFR complexes that may trigger cell death (Figure 2).

- RIPK1 was the first protein of the RIPK family identified, interacting with apoptosis antigen 1 (APO-1 or FAS) through its death domain (DD), giving it its “receptor-interacting protein” name (50). Through its DD, RIPK1 can also bind other receptors such as the TNF-receptor 1 (TNFR1), TNF-related apoptosis-inducing ligand (TRAIL) receptors 1 and 2 (DR4 and DR5) and death receptor 3 (DR3 or TRAMP). RIPK1 interacts with other adaptor proteins such as TRADD, Fas-associated protein with death domain (FADD), Toll/IL-1 receptor domain-containing adaptor inducing interferon-β (TRIF), RIP-associated ICH-1 (ICE (interleukin-1β-converting enzyme)/CED-3 homolog 1) protein with a death domain (RAIDD), TNF receptor associated factor (TRAF)1, TRAF2, TRAF3, and A20 (50–60). Furthermore, it can also interact with RIPK3, through its RIP homotypic interaction motif (RHIM) domain, as well as with focal adhesion kinase, MEKK1 and MEKK3 (61–64).

- RIPK3: similar to RIPK1; it contains an N-terminal kinase domain and a RHIM domain in the C-terminal part, that allows RIPK1/RIPK3 interactions (4). However, its C-terminal domain is completely different from other RIPK proteins. This could explain its ability to interact with the liver glycogen phosphorylase (PYGL), glutamate ammonia ligase (GLUL) and glutamate dehydrogenase 1 (GLUD1), enhancing its enzymatic activity (4). These are metabolic enzymes required for ATP production, with PYGL releasing glucose-1-phosphate from liver glycogen, and GLUL and GLUD1 playing crucial roles in the use of glutamate and glutamine as substrates for ATP through oxidative phosphorylation; this suggests a link between RIPK3 and metabolism (65). In fact, RIPK3 orchestrates necroptosis, “an active cell death pathway” that requires both adenosine triphosphate (ATP) and ROS (66). Through RIPK3-enhanced aerobic respiration, mitochondria could both produce energy to execute necroptosis while increasing the amounts of ROS required for the RIPK1/RIPK3 and later MLKL (mixed lineage kinase domain-like) complex formation and activation downstream of TNF (66).

Genetic studies has helped to understand the role of these kinases in cell death. Mice lacking RIPK1 show defects in multiple tissues, triggering systemic inflammation leading to perinatal death 1-3 days after birth (67). Simultaneous deletion of TNFR1 prolonged up to 12 days post-delivery the survival in *Ripk1*^−/−^ *Tnfr1*^−/−^ (68). Deletion of RIPK3, mixed lineage kinase domain like pseudokinase (MLKL) or caspase-8 in *Ripk1*^−/−^ did not improve the phenotype, with those mice dying soon after delivery (69, 70), suggesting that when just apoptosis or necroptosis are blocked downstream of TNF, the other pathway gets activated. In a similar manner, triple deletion of RIPK1, RIPK3, and TNFR1 allows mice survive until adulthood. Yet, shortly after birth they present intestinal apoptosis, which could contribute to the mortality associated with blood bacteremia (69). Accordingly, simultaneous deletion of RIPK1, RIPK3, and caspase-8 or FADD protect the mice and prevented any macroscopic and microscopic signs of intestinal pathology, but mice developed autoimmune lymphoproliferative syndrome (69, 70). Since double deletion of FADD and RIPK1 induces perinatal death (71) but additional deletion of RIPK3 protect the animals, these results suggest that some other mechanism, independent of RIPK1, activates RIPK3. In fact, DNA-dependent activator of interferon regulatory factors (DAI) can interact and activate RIPK3 independently of RIPK1 (72). *Ripk1*^−/−^ *Ripk*^−/−^ and *Trif*^−/−^ or *Ifnar*^−/−^ animals were generated, and although it conferred certain protection compared with *Ripk1*^−/−^ *Ripk*^−/−^, those mice did not survive past weaning (70). Finally, mice lacking RIPK1 in IECs specifically (RIPK1^Δ*IEC*^), develop severe intestinal inflammation associated with IEC apoptosis leading to early post-birth death. Similarly, tamoxifen-induced deletion of *Ripk1* leads to rapid weight loss and mice death. Crypt cells from RIPK1^Δ*IEC*^ failed to grow into organoids (69), so *in vitro* deletion is required to grow RIPK1 deficient intestinal organoids (73). Unexpectedly, in those intestinal organoids the NF-κB pathway remained intact downstream of TNF, although they undergo massive cell death (73).

Two mice models with point mutations in the kinase domain were generated to study the kinase role of RIPK1 without compromising its scaffold function. RIPK1^K45A^ and RIPK1^D138N^ (74, 75) mice were born at expected Mendelian ratios and showed no abnormalities, pointing out the importance of RIPK1 function as a scaffold protein. Fibroblasts and macrophages derived from these mice were stimulated with TNF and were shown to be protected from cell death demonstrating the role of the kinase domain from RIPK1 in triggering cell death (74).

Unlike RIPK1^−/−^, RIPK3^−/−^ mice are indistinguible from their littermates and exhibit normal downstream pathway activation from TNFR1 and TLRs (76). However, knock-in mice harboring a kinase death form of RIPK3 (RIPK3^D161N^) die at embryonic day 11.5 due to high amounts of cell death in the yolk sac vasculature. The authors of this study show how this cell death was dependent on caspase-8. Kinase death RIPK3, but not wild-type RIPK3, interacted with FADD, RIPK1, and caspase-8. Similarly, expression of RIPK3^D161N^ in the adult intestine also led to diarrhea and massive weight loss due to caspase activation, and downstream apoptosis of IECs (77). Similar results were obtained when a RIPK3 inhibitor was given to mice (78). On the contrary, another RIPK3 kinase death animal line (RIPK3^K51A^) did not present any embryonic abnormalities, and the mice were shown to be viable, fertile, and immunocompetent, as well as able to rescue the embryonic lethality seen in caspase-8 knock-out mice (78). However, RIPK3 inhibitors still induced apoptosis on cells expressing the RIPK3^K15A^. Altogether, this data suggests that RIPK3 inhibition through small molecules or the presence of the D161N mutation induces conformational changes in RIPK3 that promote apoptosis. Although those results could be secondary to a change in the RIPK3 structure due to the D161N mutation, they suggest that RIPK3 kinase inhibition leads to apoptosis.

### RIPK1 Functions in TNFRI Complex I Downstream of TNF

*TNF* is one of numerous genes implicated in IBD pathogenesis stimulated by the nuclear factor kappa-light-chain-enhancer of activated B cells (NF-κB). It codes for the prototypical inflammatory cytokine tumor necrosis factor (TNF), which has various functions in the intestine (79). TNF is synthesized as a transmembrane protein that forms homotrimeric structures, and is cleaved by a disintegrin and metalloprotease domain 17 (ADAM17) or by TNF-converting enzyme (or TACE), which releases its soluble form (80). TNF is able to bind two receptors: TNFR1 and TNFR2, which differ in their structure and expression pattern, as well as in the signaling pathways that they induce once they are engaged (80). TNFR1 is expressed in all cell types, whereas TNFR2 is mostly restricted to immune and endothelial cells. Both receptors are able to activate the NF-κB pathway through different signaling cascades as a result of strikingly different intracellular domains. TNFR1 contains a cytoplasmic death domain (DD), which is a conserved sequence of 80 amino acids that forms a distinctive fold (81, 82) and allows the recruitment of TNFR1-associated death domain protein (TRADD). TNFR2 lacks the death domain and recruits TNFR-associated factor 1 (TRAF1) and TRAF2, rather than TRADD (82–84). Both TNFR1 and TNFR2 can lead to NF-κB activation.

**Figure 3:**
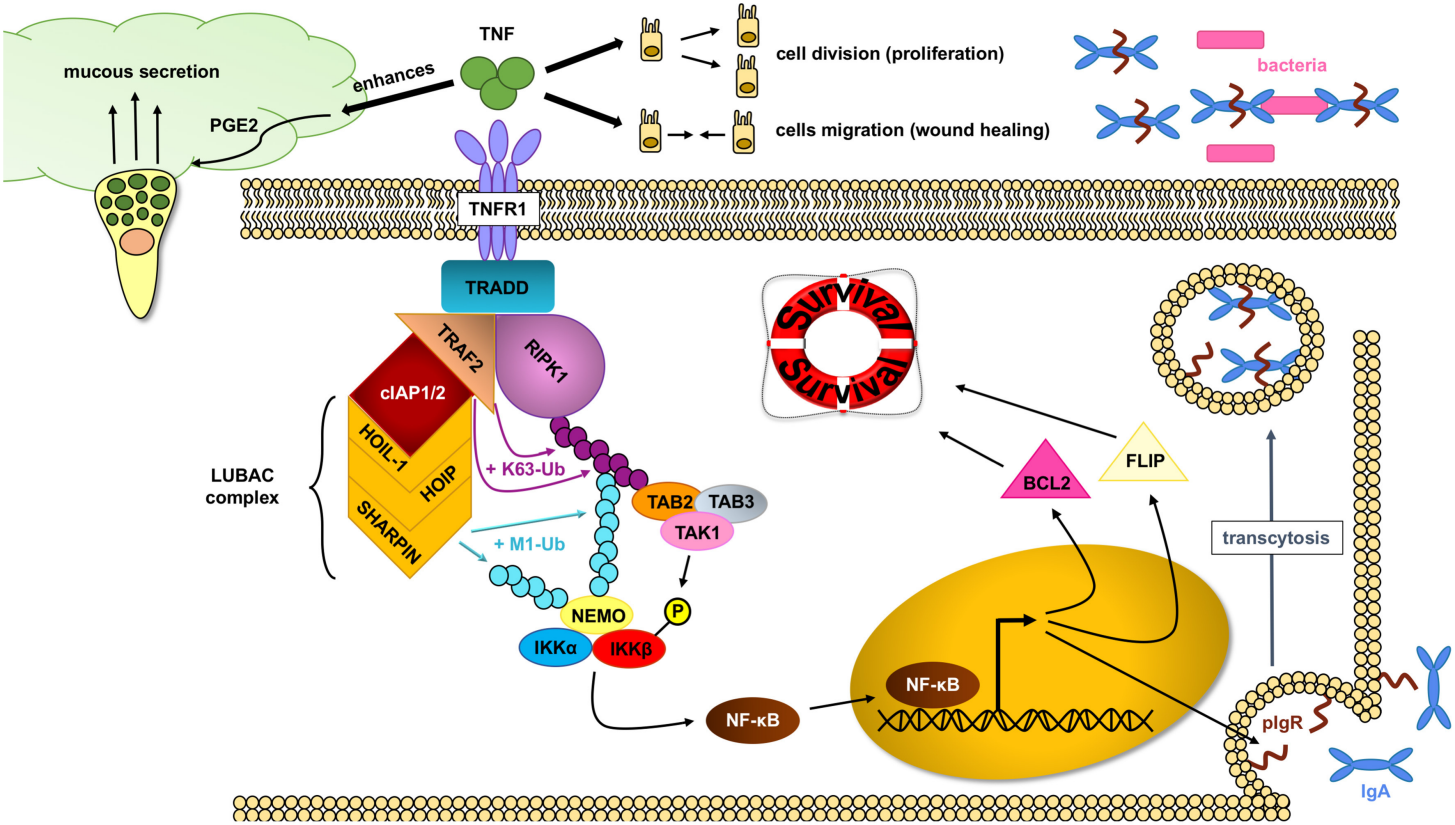
NF- κB pathway activation through RIPK1 protects IECs. In homeostatic conditions, TNF (tumor necrosis factor) plays an important role in maintaining an intact intestinal epithelial barrier. Upon binding to the TNFR1 (tumor necrosis factor receptor 1), the TNFR complex I is formed, where RIPK1 (receptor interacting protein kinase 1) serves as an scaffold protein upon which the key IKK (IκB kinase) complex will bind and activate. The IKK complex will in turn induce the translocation of the NF-κB (nuclear factor kappa-light-chain-enhancer of activated B cells) transcription factors into the nucleus and allow gene expression, including cell survival factors. TNF can also help in the wound healing process by inducing cell proliferation and migration as well as enhance the intestinal barrier by favoring mucus production and impairing bacterial translocation through pIgR (polymeric immunoglobulin receptor) induction.

TNF has important protective functions in intestinal epithelial cells (Figure 3): (a) TNF modifies the first physical barrier of the intestine: the mucus layer. Through TNFR2, TNF sensitizes goblet cells to prostaglandin E2, a known mucus secretagoge, and protects the epithelium by increasing mucus secretion (85, 86), (b) TNF is able to induce the expression of the polymeric immunoglobulin receptor (pIgR), which is necessary for the transcytosis of secretory IgA into the mucus, and prevents bacterial translocation into the lamina propria (87), (c) TNF is critical in wound healing, which is an important step in resolving injury and preventing chronification of underlying inflammation. Two different steps occur during this process: spreading and migration of cells through the basement membrane, and redifferentiation and proliferation of cells. Through TNFR2 dependent activation of focal adhesion kinase, TNF is able to induce epithelial migration (88) and cell proliferation (89). TNF can also support wound healing and cell survival through TNF-induced TACE activation, which subsequently liberates ErbB ligands that promote cell survival (90), (d) through TNFR1, TNF is able to activate the NF-κB pathway and assemble the TNFR1 complex I, which promotes IEC survival.

The assembly of the TNFR1 complex I is key in intestinal barrier maintenance. In most cell types, including IEC, transient TNF signaling inhibits apoptosis due to the assembly of the TNFR complex I, and activation of IκB kinase (IKK) β-dependent NF-κB (91). Upon the binding of TNF to homotrimers of TNFR1, the adaptor molecule TRADD is recruited to the cytoplasmatic TNFR1 domain. In a step-wise process, RIPK1, TRAF2, cellular inhibitor of apoptosis protein 1 (cIAP1) or cIAP2, and linear ubiquitin chain assembly complex (LUBAC) are recruited to form signaling complex I. TRAF2 and cIAP1/2 mediate K63-linked ubiquitination of the complex. In this situation, the kinase RIPK1 acts as a scaffold protein that allows for docking of the adaptor proteins TAK1-binding protein 2 (TAB2) and (TAB3) and the kinase TAK1 through RIPK1 K63-ubiquitins (92). Meanwhile, the LUBAC complex mediates M1-ubiquitination of some components in the complex I, such as RIPK1 and NF-kappa-B essential modulator (NEMO) (93, 94). The IKK complex is also recruited to the complex, and after phosphorylation of IKKβ by TAK1, mediates the activation of the canonical NF-κB pathway and the resulting upregulation of anti-apoptotic genes such as BCL2 (B-cell lymphoma 2) and FLIP (FLICE-like inhibitory protein), to promote cell survival and cell proliferation (80).

Several previous works have highlighted the critical role of this pathway on IEC survival. Early work by Egan et al. shows that deletion of IKKβ in IECs promotes the gut damage from ionizing radiation (IR) (95). Furthermore, when LPS, a known activator of the NF-κB pathway, is administered prior to IR, IECs are also protected from massive apoptosis, suggesting the IKK complex, the main protein complex downstream of TNF, provides protective effects to IECs (95). Similarly, IKKß was shown to be protective in a model of colitis induced by *C. difficile* and dextran sodium sulfate (DSS) (96, 97). Although deletion of IKKß or IKKß alone does not induce spontaneous colitis, IECs lacking NEMO or TAK1 develop colon pathology, in a complete or partial TNF dependent manner, respectively, including IEC apoptosis, demonstrating that the NF-κB pathway plays key homeostatic roles (98, 99). Interestingly, unlike NEMO^IEC−KO^, TAK1^IEC−KO^ develops intestinal inflammation, perhaps due to the ability of TAK1 to activate other protective pathways such as the Mitogen-activated protein kinases pathway (MAPK). Nevertheless, activated/nuclear NF-κB is present in both IECs and the lamina propria macrophages of active IBD areas (100). To determine the pathogenic function of persistent NF-κB activation, which occurs in IBD (100), we generated *Ikk*β*(EE)*^*IEC*^ mice in which a constitutively active IKKβ(EE) variant is expressed in IEC from the villin promoter (101). Surprisingly, instead of being resistant to TNF-induced mucosal erosion, *Ikk*β*(EE)*^*IEC*^ mice displayed severe TNF-dependent epithelial layer destruction when challenged with various stimuli that induce TNF production, or when given exogenous TNF (101). The mechanism by which constitutive IKKβ/NF-κB activation renders mouse IECs susceptible to TNF-induced killing rather than preventing it is unknown, but is likely to be relevant to understand the effect of NF-κB chronic activation in IECs of active IBD lesions.

### RIPK1/RIPK3 and the Assembly of TNFR Dependent Ripoptosome/Necroptosome

As reviewed above, transient TNF signaling inhibits apoptosis due to the assembly of TNFR1 complex I and IKKβ-dependent NF-κB activation (91). However, TNFR1–TRADD signaling can result in cell death in special circumstances, when complex I shifts toward complex IIa, IIb, or the necrosome to induce different types of TNF-induced cell death (Figure 2).

#### Complex IIa

Ubiquitin removal from RIPK1, through deubiquitination by cylindromatosis (CYLD), or ubiquitination-impairment by cIAP1/2 depletion (102–104), alters the formation of complex I, allowing its disassembly and TNFR1 internalization (105). TRADD, FADD, pro-caspase-8 (caspase-8), and FLICE-like inhibitory protein (FLIPs) are then recruited to the TNFR1. In this complex, the long isoform of FLIP (FLIP_L_) and the pro-caspase-8 form a heterodimeric caspase that cleaves and inactivates RIPK1 and RIPK3, as well as CYLD, to prevent necroptosis (106–108). This TRADD-dependent complex IIa also allows caspase-8 homodimerization and activation, resulting in activation of the executioners caspase-3 and caspase-7, which trigger apoptosis. This pathway, in normal conditions, would be inhibited due to previous NF-κB activation and expression of anti-apoptotic genes (108–110) but as mentioned above, ablation of IKKβ (95, 111), or its regulatory subunit NEMO (98), renders IEC susceptible to TNF-induced death.

#### Complex IIb or Ripoptosome

TNFR complex IIb or the Ripoptosome has been described to occur downstream of TNF when cIAP1/2 is depleted through SMAC mimetics (SM) (112–114). SMAC (second mitochondria-derived activator of caspase) is a pro-apoptotic mitochondrial protein that inhibits IAPs. The exact mechanism that triggers the formation of complex IIb instead of IIa is unknown, although in this case the activation of NF-κB does not prevent apoptosis (115). TNF treatment together with TAK1 pharmacological inhibition also triggers RIPK1-dependent apoptosis, in a similar manner as TNF plus SM treatment, suggesting that TAK1 recruitment to cIAP1/2-ubiquitinated RIPK1 inhibits RIPK1-dependent apoptosis (115). In fact, IKKα and IKKβ, the downstream kinases of TAK1, inhibit RIPK1 in association with the Ripoptosome through direct phosphorylation of RIPK1 (116). Complex IIb, the Ripoptosome, is composed of RIPK1, FADD and caspase-8, and A20 (117). It is independent of TRAILR1/DR4, TRAILR2/DR5, and Fas/CD95 activation (112). TLR3 can also potentially induce complex IIb. TLR3 activation induces apoptotic cell death downstream of TRIF that depends on a complex formed also by RIPK1, caspase-8, and FADD, although it is unknown whether this requires RIPK1 kinase activity (118, 119). Of note, TNFR1 activation is dispensable if cell death is triggered by etoposide, a genotoxic stress inducer that also depletes cIAPs, although in this case complex IIb formation occurs 6 h after the treatment, 4 h later than when triggered downstream of TNF. Complex IIb requires the kinase activity of RIPK1 to induce cell death although the exact mechanism of its activation or its role as a kinase is unknown; in fact no targets for the kinase activity of RIPK1 have been described apart from itself (113).

#### TNF-Induced Necroptosis

Necroptosis can be triggered through different stimuli. Most studies on necroptosis have been performed after TNF, FAS, or TLRs stimulation, but it can also be triggered by intracellular events, such as viral infection through Z-DNA or Z-RNA sensing via Z-DNA binding protein 1 (ZBP1/DAI) (72). For instance, downstream of TNF, when caspases are not fully activated or their activity is blocked (ex: by viral inhibitors), the protein kinase RIPK3 is recruited and forms the necrosome, which will lead to necroptotic cell death (120, 121). Once engaged, RIPK1 and RIPK3 undergo auto and transphosphorylation leading to their activation. Interestingly, although RIPK3 can also phosphorylate RIPK1, RIPK1 does not phosphorylate RIPK3 (120). The requirement for RIPK1 and RIPK3 trans and autophosphorylation can explain the formation of RIPK1/3 amyloid structures through RIPK1 and RIPK3 RHIM domains, a required step for RIPK3 autophosphorylation (122, 123). All these signals will converge into MLKL phosphorylation and activation, and subsequent cell death (124). Phosphorylated MLKL binds to the inner leaflet of the plasma membrane and forms the necroptotic pore, executing necroptosis (125–127). Although RIPK1 can be autophosphorylated at S14/15, S20, S161, and S166, (128), only S161 has been shown to be required to induce necroptosis (129). RIPK1 phosphorylation on S89 or MK2 mediated phosphorylation of S321 impair RIPK1 mediated cell death (130, 131). Phosphorylation of S227 in RIPK3 allows the binding of RIPK3 to MLKL (124). In addition, MLKL is phosphorylated by RIPK3 at T357 and S358 residues in human, and S345, S347, and T349 residues in mouse. These phosphorylation sites are necessary for necroptosis since mutation of both sites inhibits necroptotic cell death (124, 132).

## Dysregulation of Intestinal Epithelial Cell RIPK Pathways Promotes Cell Death in IBD

The increased areas of epithelial cell death associated with IBD are especially prevalent in UC compared to CD and controls (133). This epithelial cell death increases the chances of antigen translocation and subsequent triggering of inflammatory responses (134–137). These epithelial cell deaths present features of apoptosis and necrosis. Necrosis has long been recognized as a major trigger of inflammation; as cells die, their cellular contents activate the host immune response. Apoptotic cells will also increase the intestinal permeability by overwhelming the capacity of phagocytes to clear apoptotic cells, and by preventing proper tight junction function and intestinal barrier remodeling of patches of shedding cells. Interestingly, patients with active UC, who ultimately require surgery, had higher apoptotic indices than UC patients that were receiving medication. Also, electron microscopy on rectal biopsies of patients with CD and UC compared with normal controls showed patches of necrotic cells in four out of seven CD patients (135). Notably RIPK3 is expressed at high levels in the terminal ileums of patients with CD (138).

Several IBD pathogenic factors can promote IEC cell death in these patients. Among them, genetic predisposition, disturbance of the commensal microbiota, and dysregulated immune responses can contribute to epithelial barrier defects and promote chronic inflammation in the IBD gut.

### Genetic Predispositions to IBD

Although family history is a risk factor for developing IBD, the concordance rate in monozygotic twins is only 10–15% in UC, and 30–35% in CD, suggesting that non-genetic factors might play a bigger role (139). Nonetheless, the first genome-wide association study (GWAS) for Crohn's disease, undertaken in 2005 in Japan, identified the susceptibility locus of the tumor necrosis factor super family 15 gene (*TNFSF15*) (140). Subsequently, several other studies have identified, in different ethnic cohorts, 235 genetic markers in 200 susceptibility loci (141–143). Of the 163 identified loci in the Caucasian population, 110 appear to be relevant to both CD and ulcerative colitis (*TNFAIP3, IRGM, TNFSF15*), 23 appear to be specifically related to CD (*ATG16L1, NOD2*), and 30 appear to be specifically related to UC (*IRF5, NFKB1*). IL-23R has also been shown to be related to CD in several studies (144–146), with rs1343151 and rs7517847 variants decreasing the risk of developing the disease. Although, most identified SNPs lack functional data, the identification of these genes elucidates the critical pathways in IBD pathogenesis.

- a. The first genetic risk variant identified for CD was the *NOD2* gene (147, 148). Hugo et al. found three different polymorphisms in *NOD2*; one is a frameshift mutation (L1007C) which causes a truncated protein transcript, and two are non-synonymous polymorphisms (R702W and G908R). Carriage of one copy of any risk allele confers a modestly increased risk of developing CD (2 to 4-fold). However, having two copies or a combination thereof is associated with a 20- to 40-fold increased risk. Another SNP in the autophagy gene ATG16L1, which is associated with CD, is responsible for a threonine to alanine substitution at amino acid 300 (T300A) that increases the odds ratio (OR) for CD to 1.62 in the Spanish population (149). Finally, another important gene related to autophagy is *IRGM* (immunity-related guanosine triphosphatase family M protein). It encodes a GTP-binding protein that induces autophagy and plays an important role in innate immunity against intracellular pathogens. Two flanking SNPs (rs13361189 and rs4958847) have been better associated with increased susceptibility to CD with an OR of 1.34 and 1.33; the first SNP alone also confers a small association with UC (OR: 1.16) (149).
  Impaired autophagy disturbs the function of IECs and influences the inflammatory and immune responses, ROS production, and endoplasmatic reticulum (ER) stress, promoting the occurrence and development of IBD (150–153). Furthermore, it is noted that autophagy can play a role in the release and degradation of the damage-associated molecular pattern molecules (DAMPs), contributing to the alleviation of IBD (154–156). ATG16L1 deletion also increases the chance of IECs necroptosis (47), and deletion of another autophagy protein, ATG5, results in impaired intestinal permeability and protection against *Toxoplasma gondii* infection (157). Finally, mice deficient in *Nod2* and *Atg16l1* showed Paneth cell defects and susceptibility to intestinal inflammation (158, 159). These results highlight the importance of the Paneth cell, that releases antimicrobial peptides, supports stem cells, and regulates AMP production (*Nod2*) and granule exocytosis (*Atg16l1*), in the pathogenesis of the disease. Importantly, similar phenotypes have been observed in human disease, and patients with Crohn's disease carrying the ATG16L1^T300A^ mutation showed granular abnormalities in Paneth cells (159).
  Other authors looked into the role of the NOD2 L1007insC polymorphism, which results in a frameshift mutation that generates a truncated Nod2 protein. This mutation prevents peptidoglycan and MDP-dependent activation of the NF-κB pathway, and localization of NOD2 into the plasma membrane (28, 46, 160). NOD2 L1007insC did not prevent NOD2-ATG16L1 interaction, but did prevent its localization in the plasma membrane, impairing wapping of invading bacteria by autophagosomes. Furthermore, in different human epithelial cell lines, deletion of ATG16L1 or reconstitution with the common coding variant ATG16L1^T300A^ abrogated capture and degradation of intracellular Salmonella (161, 162). Recently, Murthy et al. showed a relation between autophagy, cell death and inflammation. The authors demonstrated that caspase-3 enhances the cleavage of ATG16L1T300A, an SNP strongly associated with incidence of CD. They propose that the presence of T300A apoptotic stimuli enhances ATG16L1 cleavage, triggering cytokine production and inflammation (163). Another work has also shown how ATG16L1 prevented necroptosis in IECs (47).
- b. RIP1 and RIP3: Although no SNPs in these proteins have been associated with IBD, the effect of RIPK1 deficiency in humans was studied by Cuchet-Lourenço et al. (3). In this study, they found four patients from three unrelated consanguineous families carrying homozygous loss-of-function mutations in RIPK1. The four patients had lymphopenia, suffered from recurrent viral, bacterial and fungal infections, early-onset inflammatory bowel disease, involving the upper and lower gastrointestinal tract, and developed arthritis (3). Stimulation of skin fibroblasts with TNF and poly(I:C) *in vitro* showed similar results to those seen in mice, with impaired activation of downstream signaling pathways from TNFR1 and TLR3 and increased cell death through necroptosis.
- c. NF-κB pathway: SNPs in ubiquitously expressed genes encoding NF-κB-regulated molecules show strong association with IBD (164, 165). NF-κB stimulates transcription of numerous genes implicated in IBD pathogenesis, including *TNF*. TNF inhibition is one of the main therapeutic options in IBD (100), leading to reduced IEC apoptosis and enhanced mucosal repair (91). In IECs, transient TNF signaling inhibits apoptosis due to IKKβ-dependent NF-κB activation (91). On the other hand, corresponding ablation of IKKβ (95, 111), or its regulatory subunit NEMO (98), renders IEC susceptible to TNF-induced death. However, IKK or NF-κB deficiencies have never been reported in IBD.
- d. A20 is a NF-κB-responsive gene that is thought to be involved in negative feedback regulation of NF-κB activation in response to many proinflammatory stimuli (166, 167). A20 contains an ovarian tumor (out) domain with deubiquitinating activity (DUB) in the amino-terminal region and seven carboxy-terminal zinc finger (ZnF) domains. A20-deficient mice have a severe inflammatory phenotype, with hypersensitivity to TNF, and die prematurely due to severe multiorgan inflammation and cachexia (168). Although several reports describe that A20 terminates the NF-κB pathway through its DUB activity by breaking down the docking sites in the TNFR1 complex I, A20 knock-in mice bearing an inactivating mutation in DUB (C103A) or ZnF4 domains do not exhibit the severe inflammatory phenotype of full A20-knockout mice (169, 170), suggesting that the function of A20 to modulate the NF-κB is not dependent on its deubiquitinase activity.

Several studies have linked SNPs of *TNFAIP3*, which codes for the immunoregulatory protein A20, with susceptibility to multiple autoimmune human diseases. These diseases include systemic lupus erythematosus (SLE), rheumatoid arthritis (RA), psoriasis, type 1 diabetes, coeliac disease, Crohn's disease, coronary artery disease in type 2 diabetes, and systemic sclerosis (171). Most of the SNPs related with IBD are located in non-exon areas, implying that they most likely play a role in RNA synthesis or maturation. The minor rs5029941 (alanine to valine substitution) allele is associated with increased risk for IBD with an OR of 3.75, while the rs7753394, located upstream to the coding region, has an OR of 1.21 in heterozygotes and 1.48 in homozygotes for CD. Finally the rs2327832 allele increases the OR for UC to 1.26 (172). Interestingly, the rs6927172 variant was associated with increased A20 expression, decreased TNF levels, and non-response to anti-TNF therapy in both CD and UC (173). On the other hand, the rs6927210, rs7753394, and rs7773904 variants were linked to improved response to anti-TNF drugs (174).

Given that A20 SNPs in other diseases, such as SLE, have been related to lower expression or function (175, 176), and that A20 deletion in the whole mouse or in different compartments, including the intestine, induces spontaneous inflammation (168, 174, 177, 178), it is thought (but not proven) that SNPs in the *TNFAIP3* gene are associated with IBD decrease A20 expression. In IEC, deletion of A20 on those cells renders the mice more susceptible to the DSS colitis model with higher amounts of apoptotic cells in the epithelial colon (179). While the previous study did not show spontaneous intestinal inflammation, combined deletion of A20 in IEC and the myeloid compartment induces spontaneous colitis and ileitis with the presence of apoptotic cells in the crypt compartment (174). Additionally, overexpression of A20 in the IEC protects the intestinal epithelial barrier after LPS challenge and prevents colitis induced by DSS but not TNBS (180, 181).

However, A20s role in cell death seems to be more dependent on cell type than its NF-κB regulatory function. An A20 specific deletion in B and T cells actually protects them from FAS and TCR (T-cell receptor) induced cell death (178, 182). Also. two independent works have looked into the RNA expression of A20 in IBD. Although Arsenescu et al. found a decrease in the RNA levels of A20, as well as other typical proinflammatory markers of IBD in non-inflamed IBD tissue compared with control samples (183), Vereecke et al. found that A20 levels of non-responder patients to anti-TNF therapy was higher both before and after treatments compared to controls and responders. Accordingly, levels of A20 in patients that responded to anti-TNF drugs diminished to basal levels after therapy. These results could suggest that the upregulation of A20 is triggering intestinal inflammation. We have recently showed that A20 protein levels in UC and CD are increased in IECs. Using transgenic mice that overexpress A20 in the IEC, we showed that increased and prolonged recruitment of A20 to the TNFR complex I favors a shift from complex I toward complex IIb, probably through maintenance of RIPK1 linear-poly-ubiquitinated status and inducing RIPK1-dependent apoptosis in IEC (117). Of interest, we also detected A20 in the ripoptsome complex (117). Concomitant with that, pharmacological and genetic RIPK1 kinase blockade prevented apoptosis, suggesting a new therapeutical treatment for IBD.

### Microbiome

The gut microbiome, including bacteria, fungi, virus, and other organisms, shapes host functions in both normal and disease conditions. The clinical observation that antibiotics have a modest effect in IBD (184–187) suggests that the microbiome could play a role in shaping the disease. In fact, bacterial dysbiosis has been shown to occur in IBD (188–191) with consistent reports of decreased biodiversity, both α diversity and species richness, a measure of the total number of species in a community. However, a specific role of bacterial dysbiosis in IBD is yet to be discovered. In fact, a recent paper by Halfvarson showed that inflammation was not directly correlated with increased dysbiosis (188). A similar concept was suggested by another study, which shows that there is reduced diversity in inflamed vs. non-inflamed tissues within the same patient, and a lower bacterial load in inflamed regions in CD patients (192). Also, serum reactivity against selected components of the gut microbiota is common, even in healthy individuals, and some CD associated serological markers against microbial antigens are present years before clinical manifestations in patients with CD, as well as in healthy individuals (193).

Various microorganisms that supposedly exert aggressive or protective functions relevant to Crohn's disease, such as adherent-invasive *Escherichia coli* and *Faecalibacterium prausnitzii*, respectively have been identified (194, 195). Furthermore, it is known that *Helicobacter pilori* has developed different mechanisms to disrupt the intracellular adhesions of the intestinal barrier (196), suggesting that other bacteria could act similarly. Yet, *E. coli* Nissle 1917 or ECOR63 enhance the epithelial barrier by up-regulating ZO-1 and claudin-14 and by downregulating claudin-2 (197). Also, Chelakkot et al. have demonstrated recently, that *Akkermancia muciniphila*, a known beneficial bacteria that reduces gut barrier disruption, upregulates occludin-2, decreasing the permeability of lipopolysaccharide-treated Caco-2 cells (198); a similar effect is seen when treating T84 monolayers with metabolites from the probiotic *Bifidobacterium infantis* Y1, which leads to an increase of ZO-1 while reducing claudin-2 (199). Treating Caco-2 cells with another probiotic, *Lactobacillus plantarum* MB452, also increased the transcription of occludins (200) and, *in vivo*, it increased occludin and ZO-1 (201). Some probiotics and commensals have also been shown to prevent, and even reverse, the adverse effects of pathogens on intestinal barrier function. For instance, when *L. plantarum* is co-cultured with enteroinvasive or enteropathogenic *E. coli*, it prevents the loss of permeability induced by those strains (202, 203). This data suggest that bacteria can directly regulate gut permeability by modulating cell-to-cell junctions. Thus, although it is believed that an inappropriate response against commensal gut microbiota occurs in IBD, it has been difficult to determine whether or not this process is secondary to an altered microbiota, a defective immune response, or a change in gut permeability (204), and whether these microbiome changes are primary or secondary to the disease. Of interest, some of the genes related to IBD were shown to control the bacterial microbiome and gut permeability, modulating cell-to-cell junctions. For instance, Nod2 prevents inflammation of the small intestine by restricting the expansion of the commensal *bacteroides vulgatus* (205).

Fungi is also a constituent of gut microbiota, however it just accounts for <0.1% of the total microbes (206). Antibiotic treatment increases fungi while decreasing bacteria populations, showing a competition between both kingdoms (207, 208). Alterations of GI bacterial populations and increased yeast can drive the development of a CD4 T-cell-mediated allergic airway response to subsequent mold spore exposure, suggesting a role for fungal microbiota in promoting immune-mediated diseases (208). In IBD patients, *Basidiomycota, Ascomycota*, and *C. albicans* are significantly elevated, whereas Saccharomyces, Candida, and Cladosporium are predominant in healthy individuals (209–211). Different components of the fungal cell wall such as chitin, β-glucans, and mannans can trigger the innate immune response, so it is not surprising that intestinal fungal invasion exacerbates colitis in mice (212).

Although virology focuses on pathogenic strains, most viruses are bacteriophages or endogenous retroviral elements. In fact, 99% of the annotated DNA viruses are bacteriophages (213). There are approximately 10^8^-10^9^ virus-like particles (VLP) per gram of human stool, suggesting that viruses could play an important role in the bacterial community. The human gut bacteriophage varies intensively between subjects. However, they are temporally stable within individuals with dsDNA *Caudovirales* and ssDNA *Microviridae*, the two predominant viruses in healthy humans (214, 215). In IBD, virome richness is increased with expansion from the order *Caudovirales* (216–218). This could be explained as a result of commensal microbes entering lytic cycles, or from new viruses introduction from new bacteria. In either case, bacteriophages can shape the gut microbiome, affecting bacterial fitness, diversity, and perhaps aiding in horizontal gene transfer (219, 220). Furthermore, viruses can translocate into the host, inducing immune responses (221–223). It is not surprising that mice with a genetic predisposition for CD (a mutation in the ATG16L1 gene) manifest the disease when infected with a gut norovirus while wild type mice controls did not (224). Another study showed that mice that were administered a cocktail of antiviral drugs had more severe colitis in the dextran sulfate sodium (DSS) model than ones treated with DSS alone. Overall, these results suggest a role for the virome in IBD, and new research will be needed to further understand its impact.

## Conclusions

**Figure 4:**
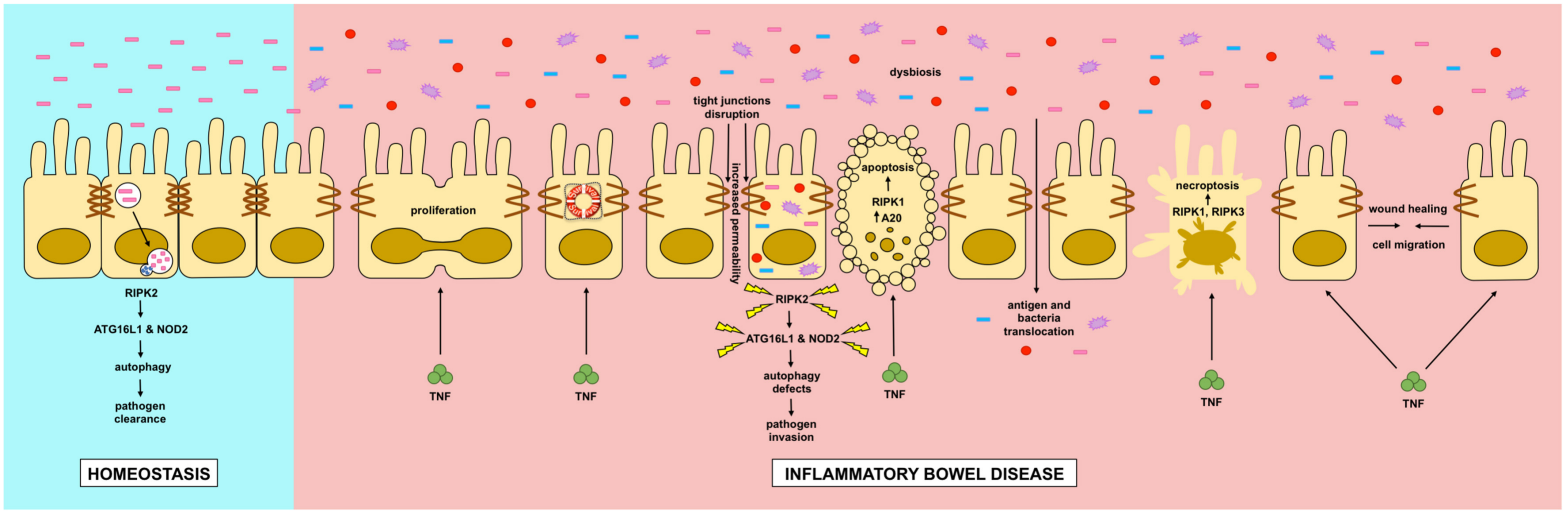
Dysregulation of intestinal epithelial cell RIPK pathways promotes cell death in IBD. Dysregulation of RIPK (receptor interacting protein kinase) pathways play a key role in the inflammatory processes occurring in IBD (inflammatory bowel disease). TNF (tumor necrosis factor) has pleyotropic roles in intestinal epithelial cells. In homeostasis, TNF through the activation of the NF-κB (nuclear factor kappa-light-chain-enhancer of activated B cells) pathway, where RIPK1 has a scaffold function, it promotes cell proliferation, migration and survival, helping to a proper intestinal barrier regeneration. In IBD, TNF can induce apoptosis or necroptosis in a kinase dependent function of RIPK1 and RIPK1/3 respectively. Cell-to-cell adhesions are also loosened in IBD, allowing the translocation of food antigens and bacteria from the gut lumen. Another feature of IBD is microbial dysbiosis. Genetic mutations in RIPK2, NOD2 (nucleotide-binding oligomerization domain-containing protein 2) or ATG16L1 (autophagy-related protein 16 like 1) can impair a proper autophagy response allowing the proliferation and invasion of the host by pathogenic bacteria.

In this review we discussed the role of RIPK and autophagy in relation to IBD (Figure 4). RIPK proteins seem to be plausible candidates for new drugs to treat inflammatory flares of IBD, preventing breakdown of the intestinal epithelial barrier. Additionally, autophagy seems to be a protective pathway, mainly by regulating intestinal homeostasis and pathogen protection, especially through paneth cells. Although further research is required to completely understand the pathophysiology of IBD, great advances in the field have improved the wellbeing of patients with the disease.

## Author Contributions

RG-C, S-JY, SD, and MG contributed to the literature review. All authors were involved in drafting the article or revising it critically for important intellectual content, and all authors approved the final version to be published.

### Conflict of Interest Statement

The authors declare that the research was conducted in the absence of any commercial or financial relationships that could be construed as a potential conflict of interest.
